# Correlation exploration among CT imaging, pathology and genotype of pulmonary ground‐glass opacity

**DOI:** 10.1111/jcmm.17797

**Published:** 2023-06-20

**Authors:** Yong Cai, Tong Chen, Shiju Zhang, Min Tan, Jiying Wang

**Affiliations:** ^1^ Department of Radiation Oncology Shanghai Tianyou Hospital Shanghai China; ^2^ Department of Radiation Oncology Shanghai Pulmonary Hospital Tongji University School of Medicine Shanghai China; ^3^ Department of Respiratory Shanghai Tenth's People Hospital Tongji University School of Medicine Shanghai China

**Keywords:** ground‐glass opacity (GGO), lung/pathology, oncogenes, tomography, X‐ray computed

## Abstract

To analyse the clinical features, imaging manifestation, pathological typing and genetic testing results of patients undergoing surgery for ground‐glass opacity (GGO) nodules, and explore the reasonable diagnosis and treatment program for GGO patients as to provide the basis for the establishment of GGO treatment process. This study is an exploratory study. 465 cases with GGO confirmed by HRCT, undergoing surgery and approved by pathologic diagnosis in Shanghai pulmonary hospital were enrolled in this study. All the patients with GGO were cases with single lesion. The relationship between the clinical, imaging, pathological and molecular biological data of single GGO were statistically studied. (1) Among 465 cases, the median age was 58 years and females were 315 (67.7%); there were 397 (85.4%) non‐smoking, and 354 cases (76.1%) had no clinical symptoms. There were 33 cases of benign and 432 cases of malignant GGO. Significant differences were observed on the size, vacuole sign, pleural indentation and blood vessel sign of GGO between two groups (*p* < 0.05). Of 230 mGGO, there were no AAH, 13 cases of AIS, 25 cases of MIA and 173 cases of invasive adenocarcinoma. The probability of solid nodules in invasive adenocarcinoma was higher than that in micro invasive carcinoma, and the difference was statistically significant (*p* < 0.05). 360 cases were followed up with the average follow‐up time of 6.05 months, and GGO of 34 cases (9.4%) increased. (2) In 428 adenocarcinoma samples approved by pathologic diagnosis, there were 262 (61.2%) lesions of EGFR mutation, 14 (3.3%) lesions of KRAS mutation, 1 (0.2%) lesion of Braf mutation, 9 (2.1%) lesions of EML4‐ALK gene fusion and 2 (0.5%) lesions of ROS1 fusion. The detection rate of gene mutation in mGGO was higher than that of pGGO. During the follow‐up period, genetic testing results of 32 GGO showed that EGFR mutation rate was 53.1%, ALK positive rate of 6.3%, KRAS mutation rate of 3.1% and no ros1 and BRAF gene mutation. No statistically significant difference was observed in comparison with unchanged GGO. (3) EGFR mutation rate of invasive adenocarcinoma was the highest (168/228, 73.7%), mainly in the 19Del and L858R point mutations. No KRAS mutation was found in atypical adenoma hyperplasia. No significant difference was observed on the mutation rate of KRAS between different types of GGO (*p* = 0.811). EML4‐ALK fusion gene was mainly detected in invasive adenocarcinoma (7/9). GGO tends to occur in young, non‐smoking women. The size of GGO is related to the degree of malignancy. Pleural depression sign, vacuole sign and vascular cluster sign are all characteristic images of malignant GGO. pGGO and mGGO reflect the pathological development of GGO. During the follow‐up, it is found that GGO increases and solid components appear, which is the indication of surgical resection. The detection rate of EGFR mutations in mGGO and invasive adenocarcinoma is high. pGGO has heterogeneity in imaging, pathology and molecular biology. Heterogeneity research helps to formulate correct individualized diagnosis and treatment plans.

## 1 INTRODUCTION

Since the ground‐glass opacity (GGO) was discovered by CT in the 1990s, with the widespread application of HRCT in lung cancer screening, the detection of pulmonary GGO has continued to increase in recent years.^1^ GGO is defined as an area of indistinct dense opacity on HRCT in which bronchial or pulmonary vascular structures are still visible, with a maximum diameter of less than 3 cm. Focal pulmonary GGO is thought to be closely associated with lung adenocarcinoma.^2^ How to properly handle GGO and make timely diagnosis and reasonable treatment for early stage lung cancer has become one of the hot spots in clinical oncology.

Identifying benign and malignant by GGO imaging features, formulating diagnosis and treatment or follow‐up strategies, and judging and grasping the timing of surgery are the key and difficult points. Lung cancer is identified from GGO and early intervention is very important to reduce lung cancer mortality. The progression of malignant GGO undergoes a continuous morphological change, and the size change, shape, lobulation, burr, air bronchus sign, etc. can be observed from continuous imaging examinations; so regular follow‐up of GGO patients is particularly important.^3^, ^4^

The International Association for the Study of Lung Cancer, the American Thoracic Society and the European Respiratory Society jointly published a new international multidisciplinary classification of lung adenocarcinoma in the Journal of Thoracic Oncology in 2011 (hereinafter referred to as the new classification of lung adenocarcinoma).^5^ The new classification reorganizes the classification system according to the linear relationship between the occurrence and development of lung adenocarcinoma, from atypical adenomatous hyperplasia, adenocarcinoma in situ (AIS), minimally invasive adenocarcinoma (MIA) to invasive adenocarcinoma (IA) and variants of IA. The use of the new classification is an important basis for establishing an individualized surgical treatment strategy for GGO. The combination of imaging features and pathological features, a deep understanding of the molecular biological characteristics of a series of special types of lung cancer such as AAH‐AIS‐MIA, is an important part of the research and establishment of GGO diagnosis and treatment strategies, and is also the focus of this study.

The molecular biology of GGO is a research hotspot in recent years, and the detection of lung cancer driver genes has attracted the attention and discussion of many scholars in the field of GGO. Several trials have shown that non‐mucinous tumours, especially in adenocarcinomas with pathological types of papillary, micropapillary and adherent growth characteristics, have a high EGFR mutation rate, and the EGFR mutation rate in AIS/MIA/LPA can even be as high as 45%.^6^, ^7^, ^8^ However, the mechanism of EGFR mutation in GGO lesions is still unclear. Some studies have confirmed that adherent tumours with GGO on imaging are prone to EGFR mutation, and the EGFR mutation rate tends to be related to the volume percentage of GGO, that is, the proportion of GGO in the lesion. The larger the ratio, the higher the EGFR mutation rate.^9^, ^10^ However, some studies have shown that EGFR mutations have nothing to do with GGO components.^11^, ^12^, ^13^ More research is needed to confirm this. Based on the above studies, the relationship between GGO imaging, pathology, and molecular biology is the key to establishing a GGO diagnosis and treatment strategy, which requires in‐depth research.

The detection rate of GGO is increasing year by year with the advancement of imaging technology and the popularisation of lung cancer screening. GGO can be a similar manifestation of many different diseases, but the prognosis of benign and malignant GGO is different. Early and reasonable treatment of malignant lesions can significantly improve the prognosis of patients. Large‐scale studies have shown that patients with AIS and MIA have a very low rate of lymph node metastasis, and if they receive radical surgery, their disease‐free survival rate can be 100% or close to 100%.^14^ Combined with HRCT imaging features, the application of new pathological classification and molecular biology detection are helpful for differential diagnosis and individualized treatment of GGO. This research topic takes GGO patients as the research object, and comprehensively evaluates through imaging, pathology, molecular biology and other means, jointly detects and analyses EGFR, KRAS, ALK fusion genes, ROS1 and BRAF genes, and analyses and studies the heterogeneity of multiple GGOs, to provide a reasonable solution for the follow‐up and diagnosis and treatment of GGO, and ultimately serve the clinic and benefit the patients.

The purpose of this study is to analyse the clinical features, imaging manifestation, pathological typing and genetic testing results of patients undergoing surgery for ground‐glass opacity (GGO) nodules, and explore the reasonable diagnosis and treatment program for GGO patients as to provide the basis for the establishment of GGO treatment process.

## 2 MATERIALS AND METHODS

### 2.1 The relationship between GGO imaging features and pathological classification

A total of 465 patients with single GGO on HRCT in Shanghai Pulmonary Hospital from January 2013 to June 2015 who underwent surgery and finally obtained pathological diagnosis were collected. Inclusion criteria: (1) received at least one HRCT examination in our hospital before surgery and found a single GGO; (2) received surgery and obtained pathological evidence; (3) patients with lung adenocarcinoma received genetic testing after surgery. The 465 patients with single GGO ranged from 24 to 82 years old, with a median age of 58 years. There were 150 males (32.3%) and 315 females (67.7%), 68 had a history of smoking (14.6%) and 397 had no smoking history. (85.4%), 111 (23.9%) patients had chest pain, cough, expectoration and other symptoms and 354 (76.1%) patients had no clinical symptoms. The clinical data of the patients (sex, age, smoking history, preoperative symptoms, etc.) are shown in Table 1. The time of first discovery of GGO and operation time of patients were recorded. 105 patients were diagnosed with GGO by HRCT for the first time, and the clinician considered that the possibility of malignancy was high, and they underwent surgery immediately after the consent of the patients. The remaining 360 patients underwent surgery after follow‐up, and the follow‐up time ranged from 1 to 74 months (The median follow‐up time was 1 month, and the mean follow‐up time was 6.05 months).

**TABLE 1:** Analysis of clinical data of benign and malignant GGO.

	Benign (*n* = 33)	Malignant (*n* = 432)	χ^2^	*p*
Age				
<65	23 (69.7%)	325 (75.2%)	0.499	0.480
≥ 65	10 (30.3%)	107 (24.8%)		
Gender				
Male	16 (48.5%)	134 (31.0%)	4.280	**0.039**
Female	17 (51.5%)	298 (69.0%)		
Smoking history				
No	27 (81.8%)	370 (85.6%)	0.360	0.548
Yes	6 (18.2%)	62 (14.4%)		
Clinical symptoms				
No	25 (75.8%)	329 (76.2%)	0.003	0.959
Yes	8 (24.2%)	103 (23.8%)		
GGO location				
Upper left lobe	9 (27.3%)	121 (28.0%)	1.654	0.799
Lower left lobe	6 (18.2%)	61 (14.1%)		
Upper right lobe	10 (30.3%)	157 (36.3%)		
Right middle lobe	1 (3.0%)	25 (5.8%)		
Lower right lobe	7 (21.2%)	68 (15.7%)		

Bold values are statistical significance *p* < 0.05.

Multi‐slice spiral CT (Siemens SOMATOM Definition AS+ 64) was used. Patient supine position, scan range from sternoclavicular joint as baseline to lung base, 120 KV, 80 mA, inspiratory phase scan, slice thickness 1 mm, pitch 1 mm, reconstruction slice thickness 5–10 mm, slice interval 5–10 mm, intravenous iodine contrast agent 100 mL; three‐dimensional reconstruction. The time of each follow‐up and imaging results were recorded until the patient underwent surgery. All CT images were observed in the lung window with a width of 1200HU and a window level of −450HU; the mediastinal window with a window width of 400HU and a window level of 40HU. Observation content: lesion location, size (maximum diameter), shape (round/sub‐round, irregular), edge (lobular, burr), internal characteristic structure (vacuole sign), relationship with surrounding tissues (respectively), relationship with the pleura and blood vessels, whether it contains solid components, etc.

According to the postoperative pathological results of GGO, they were divided into benign group and malignant group. The clinical characteristics and last preoperative imaging signs of GGO patients were analysed and compared between the two groups. The imaging differences among AAH, AIS, MIA and IA in the new classification of adenocarcinoma were focused on statistical analysis. The 360 patients who underwent surgery were followed up, and the morphological changes of GGO were dynamically observed through continuous preoperative imaging examinations, and the relationship between the morphological changes of GGO and postoperative pathology of GGO was further analysed, so as to provide a basis for the follow‐up of GGO.

### 2.2 The relationship between GGO imaging features and molecular biological features

Patients with single GGO confirmed by HRCT in Shanghai Pulmonary Hospital from January 2013 to June 2015, who underwent surgery and finally obtained the pathological diagnosis of adenocarcinoma were collected. Inclusion criteria: (1) received at least one HRCT examination in our hospital before surgery; (2) single GGO; (3) received surgery to obtain the pathological evidence of lung adenocarcinoma; (4) postoperative specimens received EGFR, KRAS, ALK, ROS1, BRAF and other five gene detection. Finally, 428 patients who met the criteria were included in the study. There were 132 males (30.8%) and 296 females (69.2%), ranging in age from 24 to 82 years old, with a median age of 58 years. 60 patients (14%) had a history of smoking, 368 cases (86%) had no smoking history, 101 cases (23.6%) had chest pain, cough, expectoration and other symptoms, and 327 cases (76.4%) had no clinical symptoms, which were found by physical examination. The time of initial GGO discovery and operation time were recorded. Of the 428 patients with lung adenocarcinoma, 94 patients underwent surgery immediately after the initial HRCT discovery of GGO and 334 patients underwent surgery after follow‐up, ranging from 1 to 74 months (median follow‐up time 1 months, the mean follow‐up time was 5.87 months).

HRCT examination equipment and image collection were the same as in the previous part of the experiment. Each gene was detected using a gene detection kit from Beijing Yakangbo Biotechnology Co., Ltd., and the detection principle was real‐time fluorescent PCR. DNA was extracted from the specimen for detection of EGFR, KRAS, BRAF, and RNA was extracted for detection of ALK and ROS1.

To analyse the relationship between the last imaging features before surgery and the results of genetic testing of postoperative specimens in GGO patients who were surgically resected and confirmed by pathology as adenocarcinoma. The 334 patients who underwent surgery after follow‐up were dynamically observed the morphological changes of GGO through continuous preoperative imaging examinations, and further analysed the relationship between the morphological changes of GGO and the results of genetic testing of GGO postoperative specimens.

### 2.3 The relationship between GGO pathological types and molecular biological features

Patients with single GGO confirmed by HRCT in Shanghai Pulmonary Hospital from January 2013 to June 2015, who underwent surgery and finally obtained the pathological diagnosis of adenocarcinoma were collected. Inclusion criteria: (1) received at least one HRCT examination in our hospital before surgery; (2) single GGO; (3) underwent surgery to obtain the pathological evidence of lung adenocarcinoma; (4) received genetic testing in postoperative specimens. Finally, 428 patients who met the criteria were included in the study. There were 132 males (30.8%) and 296 females (69.2%), ranging in age from 24 to 82 years old, with a median age of 58 years. 60 patients (14%) had a history of smoking, 101 (23.6%) patients had chest pain, cough, expectoration and other symptoms and 327 (76.4%) patients had no clinical symptoms, which were found by physical examination. According to the new classification of lung adenocarcinoma, 428 cases of GGO were divided into 11 (2.6%) atypical adenomatous hyperplasia, 103 (24.1%) adenocarcinoma in situ, 86 (20.1%) minimally invasive adenocarcinoma and invasive adenocarcinoma. 228 (53.3%). The postoperative specimens were tested for five genes including EGFR, BRAF, KRAS, ALK and ROS1.

The genetic testing method is the same as the previous experimental part. To analyse the relationship between the GGO pathological type of adenocarcinoma confirmed by surgery and pathology and the results of postoperative genetic testing.

### 2.4 Statistical analysis

SPSS13.0 software was used for statistical analysis. Chi‐square test or Fisher's exact test was used for univariate analysis. *p* < 0.05 was considered statistically significant.

## 3 RESULTS

### 3.1 The relationship between GGO imaging features and pathological classification

#### 3.1.1 GGO pathological classification and characteristics

**FIGURE 1:**
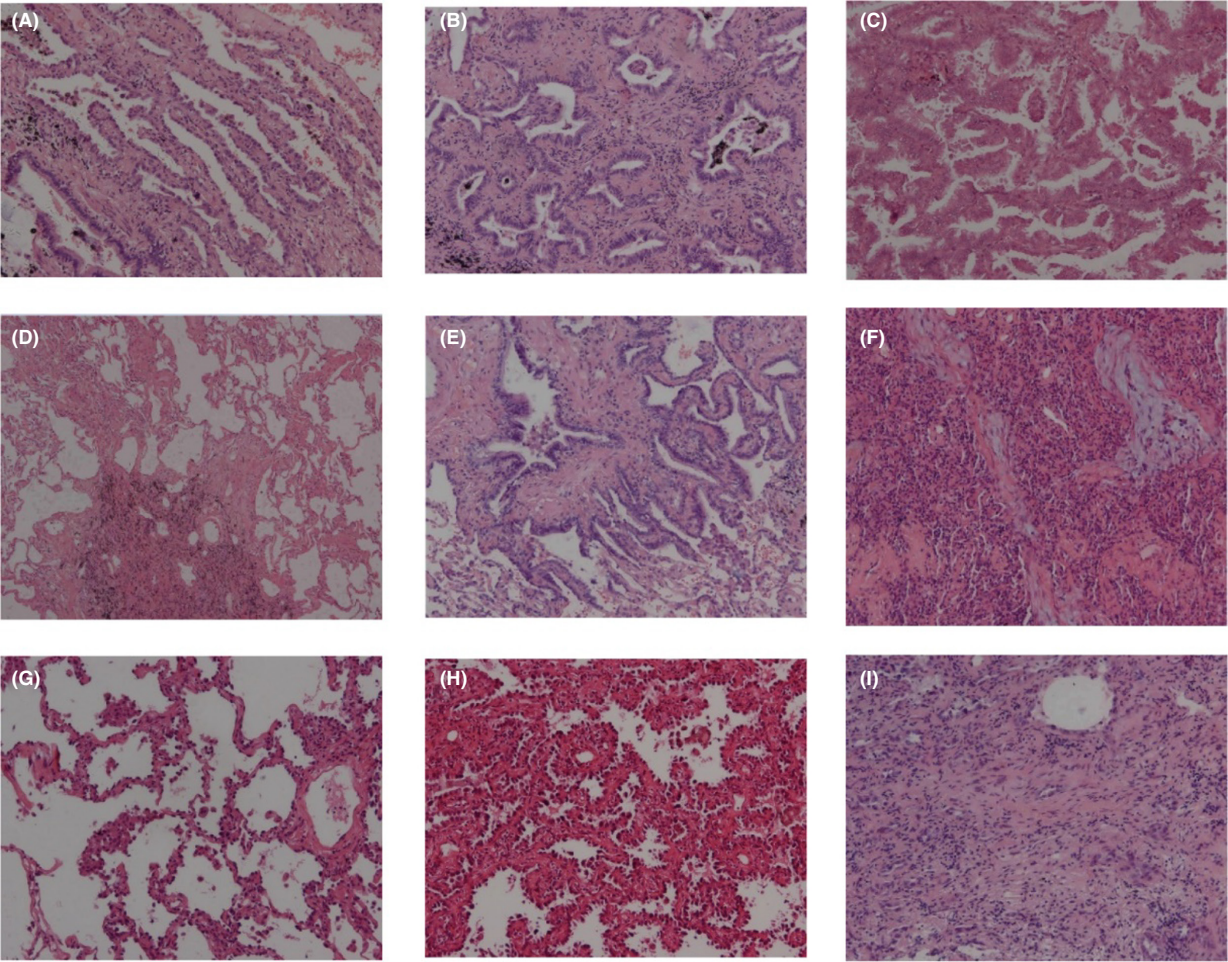
(A) Fibrofocal hyperplasia. (B) Alveolar epithelial hyperplasia. (C) organising pneumonia. (D) AAH. (E) AIS. (F) MIA. (G) LPA. (H) IA alveolar growth. (I) IA papillary growth.

GGO is mainly divided into benign GGO and malignant GGO. Among the GGO cases in this study, benign GGO accounted for less than 10%, mainly including three subtypes of fibrofocal hyperplasia, alveolar epithelial hyperplasia and organising pneumonia. Malignant GGO accounts for more than 90%, mainly including six subtypes of AAH, AIS, MIA, LPA, IA alveolar growth and IA papillary growth. The specific pathological morphology of each subtype is shown in Figure 1.

#### 3.1.2 Clinical and imaging features of different GGO pathological groups

465 cases with single GGO were divided into benign group (33 cases) and malignant group (432 cases). The clinical data of benign GGO and malignant GGO were further analysed, and the results are shown in Table 1. The results showed that there was no significant difference in age, smoking history, clinical symptoms and the location of GGO between benign and malignant. Gender was statistically significant in the difference between benign and malignant GGO (*p* = 0.039).

Further combined with the pathological results after GGO, the preoperative imaging features of benign group (33 cases) and malignant group (432 cases) were compared and analysed. The results in Table 2 showed that the differences in GGO size, vacuole sign, pleural depression sign and vascular sign were statistically significant (*p* < 0.05).

**TABLE 2:** Preoperative imaging features of benign and malignant GGO.

	Benign (*n* = 33)	Malignant (*n* = 432)	χ^2^	*p*
Size				
<10 mm	23 (69.7%)	177 (41.0%)	10.320	**0.001**
≥ 10 mm	10 (30.3%)	255 (59.0%)		
Shape				
Round	13 (39.4%)	122 (28.2%)	1.883	0.390
Round‐shaped	13 (39.4%)	208 (48.1%)		
Irregular shape	7 (21.2%)	102 (23.6%)		
Solid ingredient				
No	17 (51.5%)	218 (50.5%)	0.014	0.907
Yes	16 (48.5%)	214 (49.5%)		
Lobes				
No	17 (51.5%)	161 (37.3%)	2.634	0.105
Yes	16 (48.5%)	271 (62.7%)		
Glitch				
No	16 (48.5%)	158 (36.6%)	1.857	0.173
Yes	17 (51.5%)	274 (63.4%)		
Vacuolation sign				
No	30 (90.9%)	327 (75.7%)	3.980	**0.046**
Yes	3 (9.1%)	105 (24.3%)		
Pleural indentation sign				
No	31 (93.9%)	324 (75.0%)	5.086	**0.024**
Yes	2 (6.1%)	108 (25.0%)		
Vascular sign				
No	26 (78.8%)	266 (61.6%)	3.888	**0.049**
Yes	7 (21.2%)	166 (38.4%)		

Bold values are statistical significance *p* < 0.05.

Among the 432 cases of malignant GGO, 428 cases were adenocarcinoma, which was the main pathological type. According to the new classification of adenocarcinoma, all GGOs whose histopathological findings were adenocarcinoma were divided into four groups: AAH (11 cases), AIS (103 cases), MIA (86 cases) and IA (228 cases). The average maximum diameter of GGO in each group was AAH: 6.27 ± 1.43 mm; AIS: 9.62 ± 4.30 mm; MIA: 11.03 ± 4.64 mm; IA: 17.63 ± 7.19 mm. The difference was statistically significant (*p* < 0.001). The imaging features of different adenocarcinoma subtypes were statistically analysed, and the results are shown in Table 3.

**TABLE 3:** Imaging comparison in different adenocarcinoma subgroups.

	AAH (*n* = 11)	AIS (*n* = 103)	MIA (*n* = 86)	IA (*n* = 228)	χ^2^	*p*
Size						
<10 mm	11 (100.0%)	77 (74.8%)	52 (60.5%)	35 (15.4%)	139.944	**0.000**
≥ 10 mm	0	26 (25.2%)	34 (39.5%)	193 (85.6%)		
Shape						
Round	8 (72.7%)	57 (55.3%)	25 (29.1%)	30 (13.2%)	80.229	**0.000**
Round‐shaped	2 (18.2%)	36 (35.0%)	46 (53.5%)	123 (53.9%)		
Irregular shape	1 (9.1%)	10 (9.7%)	15 (17.4%)	75 (32.9%)		
Solid ingredient						
No	11 (100.0%)	90 (87.4%)	61 (70.9%)	55 (24.1%)	144.647	**0.000**
Yes	0	13 (12.6%)	25 (29.1%)	173 (75.9%)		
Lobes						
No	10 (90.9%)	68 (66.0%)	38 (44.2%)	43 (18.9%)	84.873	**0.000**
Yes	1 (9.1%)	35 (34.0%)	48 (55.8%)	185 (81.1%)		
Glitch						
No	10 (90.9%)	63 (61.2%)	42 (48.8%)	40 (17.5%)	82.342	**0.000**
Yes	1 (9.1%)	40 (38.8%)	44 (51.2%)	188 (82.5%)		
Vacuolation sign						
No	11 (100.0%)	96 (93.2%)	72 (83.7%)	145 (63.6%)	41.853	**0.000**
Yes	0	7 (6.8%)	14 (16.3%)	83 (36.4%)		
Pleural indentation sign						
No	11 (100.0%)	101 (98.1%)	72 (83.7%)	136 (59.6%)	64.604	**0.000**
Yes	0	2 (1.9%)	14 (16.3%)	92 (40.4%)		
Vascular sign						
No	11 (100.0%)	82 (79.6%)	62 (72.1%)	108 (47.4%)	44.439	**0.000**
Yes	0	21 (20.4%)	24 (27.9%)	120 (52.6%)		

Bold values are statistical significance *p* < 0.05.

### 3.2 The relationship between GGO imaging features and molecular biological features

#### 3.2.1 Gene mutation of GGO patients

Among 428 GGO pathological samples with pathologically confirmed adenocarcinoma, 262 (61.2%) GGO lesions had EGFR gene mutations, including 112 exon 19 deletions, 136 L858R missense mutations, 4 20INS insertion mutations, 3 G719X mutation and 3 L861Q mutations. Two cases of GGO were found to have L858R combined with 19Del, 1 case of L858R combined with S768I and 1 case of L858R combined with T790M. 14 (3.3%) GGO lesions had KRAS mutations. 9 (2.1%) GGO lesions had EML4‐ALK gene fusions. ROS1 fusion gene was detected in 2 (0.5%) GGO lesions. 1 (0.2%) GGO fusion had BRAF mutation. Most mutations are mutually exclusive. EGFR mutation (L858R missense mutation) and ALK gene fusion coexisted in 2 GGO lesions, and EGFR mutation (L858R missense mutation) and KRAS mutation (12 codons) coexisted in 1 GGO lesion. The results of genetic testing are shown in Table 4.

**TABLE 4:** GGO genes detection results.

Genes	*N* (%)
EGFR	262 (61.2%)
19Del	112/262 (42.7%)
L858R	136/262 (51.9%)
20INS	4/262 (1.5%)
G719X	3/262 (1.1%)
L861Q	3/262 (1.1%)
L858R/19Del	2/262 (0.8%)
L858R/S768I	1/262 (0.4%)
L858R/T790M	1/262 (0.4%)
KRAS	14 (3.3%)
EML4‐ALK	9 (2.1%)
ROS1	2 (0.5%)
BRAF	1 (0.2%)

#### 3.2.2 Analysis of clinical data and gene mutation detection of GGO patients

Gender and smoking history were all correlated with the EGFR mutation rate, and the difference was statistically significant (*p* < 0.05). The EGFR mutation rate in females (193/296, 65.2%) was higher than that in males (69/132, 52.3%) (*p* = 0.011). The EGFR mutation rate in patients without smoking history (236/368, 64.1%) was higher than that in patients with smoking history (26/60, 43.3%) (*p* = 0.002). There was no significant correlation between age and preoperative symptoms and EGFR mutation. The detailed results are shown in Table 5.

**TABLE 5:** The relationship between clinical characteristics of patients and EGFR mutations.

	EGFR		χ^2^	*p*
	Negative (*n* = 166)	Positive (*n* = 262)		
Age				
<65	128 (77.1%)	194 (74.0%)	0.512	0.474
≥65	38 (22.9%)	68 (26.0%)		
Gender				
Male	63 (38.0%)	69 (26.3%)	6.428	**0.011**
Female	103 (62.0%)	193 (73.7%)		
Smoking history				
No	132 (79.5%)	236 (90.1%)	9.398	**0.002**
Yes	34 (20.5%)	26 (9.9%)		
Clinical symptoms				
No	127 (76.5%)	200 (76.3%)	0.002	0.968
Yes	39 (23.5%)	62 (23.7%)		

Bold values are statistical significance *p* < 0.05.

The positive detection of KRAS was related to the patient's age, gender and smoking history. Among the 14 KRAS positive patients, the mutation rate in males (8/132, 6.1%) was higher than that in females (6/296, 2.0%) (*p* = 0.030); the mutation rate in patients with smoking history (6/60, 10.0%) was higher than that in patients without smoking history (8/368, 2.2%) (*p* = 0.002). The detailed results are shown in Table 6.

**TABLE 6:** The relationship between clinical characteristics of patients and KRAS mutations.

	KRAS		χ^2^	*p*
	Negative (*n* = 414)	Positive (*n* = 14)		
Age				
<65	316 (76.3%)	6 (42.9%)	8.142	0.004
≥65	98 (23.7%)	8 (57.1%)		
Gender				
Male	124 (30.0%)	8 (57.1%)	4.694	0.030
Female	290 (70.0%)	6 (42.9%)		
Smoking history				
No	360 (87.0%)	8 (57.1%)	9.986	0.002
Yes	54 (13.0%)	6 (42.9%)		
Clinical symptoms				
No	315 (76.1%)	12 (85.7%)	0.696	0.404
Yes	99 (23.9%)	2 (14.3%)		

#### 3.2.3 Analysis of preoperative imaging features and gene mutation detection of GGO

According to the last imaging features before surgery, the relationship between the preoperative imaging features of GGO and positive EGFR, KRAS, ALK detection was analysed by univariate, including GGO size (maximum meridian), shape (round, round‐like, irregular shape), edge (lobed, burr), internal characteristic structure (vacuole sign), relationship with surrounding tissues (respectively with the pleura, blood vessels), whether it contains solid components, etc. The detailed results are shown in Table 7.

**TABLE 7:** The relationship between the preoperative imaging features of GGO and the results of the three major genes.

	EGFR positive (*n* = 262)	χ^2^	*p*	KRAS positive (*n* = 14)	χ^2^	*p*	ALK positive (*n* = 9)	χ^2^	*p*
Size									
<10 mm	87 (33.2%)	16.492	**0.000**	5 (35.7%)	0.160	0.689	2 (22.2%)	1.325	0.250
≥10 mm	175 (66.8%)			9 (64.3%)			7 (77.8%)		
Shape									
Round	57 (21.8%)	14.627	**0.001**	6 (42.9%)	1.756	0.416	2 (22.2%)	0.509	0.775
Round‐shaped	133 (50.8%)			6 (42.9%)			4 (44.4%)		
Irregular shape	72 (27.4%)			2 (14.2%)			3 (33.4%)		
Solid ingredient									
No	83 (31.7%)	6.016	**0.014**	6 (42.9%)	0.276	0.599	2 (22.2%)	0.779	0.377
Yes	179 (68.3%)			8 (57.1%)			7 (77.8%)		
Lobes									
No	83 (31.7%)	8.657	**0.003**	6 (42.9%)	0.202	0.653	1 (11.1%)	2.670	0.102
Yes	179 (68.3%)			8 (57.1%)			8 (88.9%)		
Glitch									
No	192 (73.3%)	2.148	0.143	8 (57.1%)	2.710	0.100	7 (77.8%)	0.022	0.883
Yes	70 (26.7%)			6 (42.9%)			2 (22.2%)		
Vacuolation sign									
No	185 (70.6%)	6.183	**0.013**	11 (78.6%)	0.111	0.739	7 (77.8%)	0.044	0.833
Yes	77 (29.4%)			3 (21.4%)			2 (22.2%)		
Pleural indentation sign									
No	144 (55.0%)	11.999	**0.001**	9 (64.3%)	0.049	0.824	7 (77.8%)	1.035	0.309
Yes	118 (45.0%)			5 (35.7%)			2 (22.2%)		
Vascular sign									
No	116 (44.3%)	11.160	**0.001**	6 (42.9%)	0.356	0.551	3 (33.3%)	1.109	0.292
Yes	146 (55.7%)			8 (57.1%)			6 (66.7%)		

Bold values are statistical significance *p* < 0.05.

#### 3.2.4 Analysis of the dynamic changes of GGO preoperative imaging and the detection of gene mutation

Of 428 patients with lung adenocarcinoma, 94 underwent surgery immediately after initial HRCT finding of GGO and 334 underwent surgery after follow‐up for 1 to 74 months (median follow‐up 1 month, mean follow‐up 5.87 months). Among them, 302 cases of GGO size did not change, and the gene detection results were EGFR mutation rate of 58.9%, KRAS mutation rate of 4.0% and ALK detection rate of 2.0%. The GGO of 32 cases increased during the follow‐up period, and the gene detection results were EGFR mutation rate of 53.1%, KRAS mutation rate of 3.1% and ALK detection rate of 6.3%, but the difference between groups was not statistically significant (*p* > 0.05). The detailed results are shown in Table 8. Both ROS1‐positive GGOs and 1 BRAF‐positive GGO did not increase the maximum diameter of GGOs during follow‐up.

**TABLE 8:** Correlation between GGO maximum diameter change and genetic testing results.

	No GGO increase (*n* = 302)	GGO increase (*n* = 32)	χ^2^	*p*
EGFR				
Negative	124 (41.1%)	15 (46.9%)	0.403	0.526
Positive	178 (58.9%)	17 (53.1%)		
KRAS				
Negative	290 (96.0%)	31 (96.9%)	0.056	0.813
Positive	12 (4.0%)	1 (3.1%)		
ALK				
Negative	296 (98.0%)	30 (93.8%)	2.249	0.134
Positive	6 (2.0%)	2 (6.3%)		

### 3.3 The relationship between GGO pathological types and molecular biological features

All the 428 GGO cases diagnosed with adenocarcinoma by surgery and pathology were divided into AAH 11 (2.6%), AIS 103 (24.1%), MIA 86 (20.1%) and IA 228 (53.3%). The different subtypes of IA were 136 cases of lepidic growth type, 48 cases of acinar growth type, 32 cases of papillary growth type, 7 cases of micropapillary type, 4 cases of solid growth type and 1 case of mucinous type. The postoperative specimens were all subjected to genetic testing, and the specific test results are as follows.

#### 3.3.1 The relationship between pathological types and gene mutation

IA had the highest EGFR mutation rate (168/228, 73.7%), followed by MIA (49/86, 57.0%). There was a significant difference between GGO adenocarcinoma types and EGFR mutation rate (χ^2^ = 38.461, *p* < 0.001). Mainly seen in 19Del and L858R point mutations. The EGFR gene mutation results of different adenocarcinoma types are shown in Table 9.

**TABLE 9:** EGFR gene mutation results in different adenocarcinoma types.

	EGFR mutation			
	Negative (*n* = 166)	19Del (*n* = 112)	L858R (*n* = 136)	Other types (*n* = 14)
AAH	6 (3.6%)	3 (2.7%)	2 (1.5%)	0
AIS	63 (38.0%)	18 (16.1%)	18 (13.2%)	4 (28.6%)
MIA	37 (22.3%)	25 (22.3%)	22 (16.2%)	2 (14.3%)
IA	60 (36.1%)	66 (58.9%)	94 (69.1%)	8 (57.1%)

Further statistical analysis of different subtypes in IA showed that the invasive adenocarcinoma with adherent growth, acinar‐like growth and papillary‐like growth had a higher EGFR mutation rate, among which 19Del and L858R point mutations were the main ones. The results are shown in Table 10.

**TABLE 10:** EGFR gene mutation results in different invasive adenocarcinoma subtypes.

	EGFR mutation			
	Negative (*n* = 60)	19Del (*n* = 66)	L858R (*n* = 94)	Other types (*n* = 8)
Lepidic type	34 (56.7%)	36 (54.5%)	62 (66.0%)	4 (50.0%)
Acinar type	13 (21.7%)	16 (24.2%)	18 (19.1%)	1 (12.5%)
Papillary type	6 (10.0%)	11 (16.7%)	12 (12.8%)	3 (37.5%)
Micropapillary type	3 (5.0%)	2 (3.0%)	2 (2.1%)	0
Solid growth type	3 (5.0%)	1 (1.6%)	0	0
Mucoid type	1 (1.6%)	0	0	0

No KRAS mutation was found in atypical adenomatous hyperplasia, and there was no significant difference between GGO adenocarcinoma types and KRAS mutation rate. The results are shown in Table 11. Of the 7 invasive adenocarcinomas with KRAS mutations, 6 were of the acinar‐like growth type and 1 was of the adherent growth type.

**TABLE 11:** KRAS gene mutation results in different adenocarcinoma types.

	KRAS mutation		χ^2^	*p*
	Negative (*n* = 414)	Positive (*n* = 14)		
AAH	11 (2.7%)	0	0.755	0.834
AIS	100 (24.1%)	3 (21.4%)		
MIA	82 (19.8%)	4 (28.6%)		
IA	221 (53.4%)	7 (50.0%)		

EML4‐ALK fusion gene was mainly detected in invasive adenocarcinoma (7/9), 3 cases were adherent growth type, 2 cases were acinar‐like growth type, 1 case was solid‐like growth type and 1 case was mucinous adenocarcinoma. No EML4‐ALK fusion gene was found in atypical adenomatous hyperplasia, and there was no significant difference between the different adenocarcinoma types of GGO and the detection rate of EML4‐ALK fusion gene. The results are shown in Table 12.

**TABLE 12:** EML4‐ALK gene mutation results in different adenocarcinoma types.

	EML4‐ALK mutation		χ^2^	*p*
	Negative (*n* = 419)	Positive (*n* = 9)		
AAH	11 (2.6%)	0	1.617	0.589
AIS	102 (24.3%)	1 (11.1%)		
MIA	85 (20.3%)	1 (11.1%)		
IA	221 (52.8%)	7 (77.8%)		

#### 3.3.2 The relationship between pathological types and ROS1 gene mutation

There were 2 cases of ROS1‐positive GGO, 1 case of adenocarcinoma in situ and 1 case of invasive adenocarcinoma with acinar‐like growth type.

#### 3.3.3 The relationship between pathological types and BRAF gene mutation

One case of BRAF‐positive GGO was an invasive adenocarcinoma with acinar‐like growth.

## 4 DISCUSSION

The detection rate of GGO is increasing year by year, and it is more likely to occur in young, female, and non‐smoking patients. Patients with focal GGO are usually not accompanied by special clinical symptoms at the time of discovery.^15^, ^16^ We use HRCT scanning to make focal GGO in the lung show more detailed CT signs, which is very helpful for qualitative diagnosis. According to the correlation study between CT images and pathological results, the maximum diameter of GGO is related to the pathological type. Signs such as ‘pleural indentation sign’, ‘vascular sign’ and ‘vacuole sign’ are more common in malignant GGO, but in practical work, Not only one of them can be used as the basis for diagnosis, but a comprehensive analysis should be carried out. pGGO and mGGO reflect the pathological development of lung adenocarcinoma GGO, which can help us in preoperative imaging diagnosis and follow‐up planning. During follow‐up, GGO was found to be enlarged, which is an indication for surgical resection.

EGFR, ALK, BRAF, KRAS, ROS1 are the five common genes in lung adenocarcinoma, and EGFR gene mutation is the most common. The dominant population is female and non‐smoking.^17^ Among the imaging features, the mGGO mutation rate was high, and the L858R point mutation was the main one, followed by the exon 19 deletion mutation.^18^ Multivariate analysis showed that the size of GGO was positively correlated with the mutation rate. It was found that KRAS mutation (codon 12) and EGFR mutation (L858R missense mutation) coexisted in one GGO lesion. More research is needed to analyse the relationship between KRAS and EGFR gene mutations in the occurrence of lung cancer. Smoking and male sex may be the predominant population for KRAS mutations, but neither reached statistical significance in multivariate logistic regression analysis. Imaging features cannot be used as a reference basis for KRAS mutation. Smoking history was a characteristic factor for EML4‐ALK detection, and imaging features were not significantly associated with its mutation rate. The positive ROS1 fusion gene is more common in young, non‐smoking lung adenocarcinoma patients and the analysis of imaging characteristics needs more research data to support. BRAF is more common in more invasive, poor‐prognosis types and in patients who smoke, and thus is less common in GGO.

The correlation study between the dynamic changes of GGO and the five most common genes of EGFR, KRAS, ALK, ROS1 and BRAF in lung adenocarcinoma showed that there was no significant correlation between the maximum diameter changes of GGO and each driver gene. The different reflections of the CT signs in the developmental stages are reflected in the morphological differences, and there is no fundamental change at the molecular level. Second, the follow‐up time is short, and the number of GGO cases with dynamic changes is small, which needs to be confirmed by further research.

The treatment of lung cancer has entered the molecular era, and NSCLC is subdivided into various molecular subtypes. With the application of the new classification of adenocarcinoma in 2011, the identification of histopathological features and driver gene detection will help patients to be diagnosed in an all‐round way. and individualized treatment.^19^, ^20^ As a special group of GGO, different histological subtypes have different biological characteristics, which is of great significance for the establishment of GGO diagnosis and treatment strategies.

Although this study adopted a sufficient sample size to verify the conclusions in the study, it did not calculate and prove the sample size when selecting the sample, which is one of the limitations of this study. In addition, although this study has obtained fairly detailed results, more experiments are still needed to carry out more in‐depth studies to explore GGO.

## 5 CONCLUSION

The detection amount of GGO is increasing year by year. The clinical characteristics and imaging characteristics of GGO cases are summarized, combined with the new histopathological classification of adenocarcinoma in 2011, and the analysis of the detection results of driver genes Neither overtreatment nor missed early treatment of lung malignancies affects prognosis. GGO is more common in young women who do not smoke. It is mostly found by physical examination and has no clinical symptoms. The size of GGO has statistical significance in the distinction between benign and malignant. The vacuolar sign, pleural depression sign, and vascular sign are all characteristic images of malignant GGO. pGGO and mGGO reflect the pathological development of GGO. EGFR gene mutation is the most common, and its dominant population is female and non‐smoking. Among the imaging features, the mGGO mutation rate was high, and the L858R point mutation was the main one, followed by the exon 19 deletion mutation. Multivariate analysis showed that the size of GGO was positively correlated with the mutation rate. No correlation between imaging features and KRAS, ALK, ROS1 and BRAF gene mutations has been found. GGO is a different reflection of the same disease in different stages of development on CT signs, which is reflected in the difference in morphology and may not have fundamental changes at the molecular level.

## AUTHOR CONTRIBUTIONS

**Yong Cai:** Conceptualization (equal); data curation (equal); formal analysis (equal); writing – review and editing (equal). **Tong Chen:** Data curation (equal); formal analysis (equal); writing – review and editing (equal). **Shiju Zhang:** Data curation (equal); formal analysis (equal); writing – review and editing (equal). **Min Tan:** Data curation (equal); formal analysis (equal); writing – review and editing (equal). **Jiying Wang:** Conceptualization (equal); data curation (equal); formal analysis (equal); writing – original draft (equal); writing – review and editing (equal).

## FUNDING INFORMATION

Medical Innovation Research Project of Shanghai Science and Technology Commission (22Y11901500).

## CONFLICT OF INTEREST STATEMENT

The authors declared that they have no competing interests.

## Data Availability

Data was available on request from the corresponding author.
